# The cultural sector in China through the lens of cultural policies concepts

**DOI:** 10.12688/openreseurope.20127.2

**Published:** 2025-10-22

**Authors:** Dorota Ilczuk, Marcin Jacoby, Tamara Kaminska

**Affiliations:** 1 Faculty of Humanities, SWPS University of Social Sciences and Humanities, Warsaw, Masovian Voivodeship, Poland

**Keywords:** Cultural and Creative Sectors, CCS, Chinese cultural sector, creative industries, Chinese cultural policy

## Abstract

**Background:**

The cultural policies of Mainland China have been subject to fascinating changes in the last forty years, influenced by politics and ideology on the one hand, and market forces on the other. The article provides a preliminary outline of the main traits of the system, analysed from the perspective of contemporary cultural policies theory and international practice.

**Method:**

The authors examine how the cultural sector of the People’s Republic of China (PRC) is organized and financed, including its governance, funding, copyright, basic cultural providers and consumers. The methodological approach used adopts the analytical framework of the Compendium of Cultural Policies & Trends, and includes analyses of statistical data, primary and secondary sources.

**Results:**

Data and source information show that cultural policies of the PRC are a function of central-level political policy-making of the party-state. Cultural activity at all levels is subject to strong political control. At the same time, there is visible tension between central-level general policy guidelines and local level implementation, and between the mission of the state to ensure wide cultural participation, and the market economy incentivization of public cultural organizations.

**Conclusions:**

The authors conclude that the sector exhibits a dual characteristics, with market-insulated public services on the one side, and the market economy Cultural and Creative Sectors (CCS) on the other, where state-owned enterprises compete for services, customers, and profits with private entities. Faced with numerous challenges and new developments, the sector also witnesses dynamic changes of its legal environment.

## Introduction

### Aim of the paper

This paper presents the results of research addressing the issue of how the People's Republic of China (PRC), excluding Hong Kong and Macao, shapes its cultural policies within the framework of contemporary cultural policies theory and international practice. The authors apply the concept of cultural policy to identify the specifics of the cultural and creative sectors (CCS) in Mainland China. The focus is on identifying issues that directly relate to how the CCS are organized and financed, including their governance, funding, copyright, basic cultural providers, and consumers. Research outcomes are presented in the form of a structured profile of China's cultural policies and include the first publicly known organigram of the country's cultural sector organization and management.

In this article, we employ the term “cultural and creative sectors” as understood within the concept of the creative economy. The concept was presented by John Howkins in 2001 in his book, *The Creative Economy. How People Make Money from Ideas* ^1^. Broadly speaking, this economy refers to activities that stem from individual creativity and talent, and which also have the potential to create GDP and jobs through the creation and use of intellectual property rights. Goods and services of subjective importance are then produced, whose symbolic value is higher than their use value. There are many definitions of the creative economy and its "mapping", i.e. indicating industries and types of activity included in it. The definition we adopt for our research is related to its cultural component that links non-industrial areas of culture (major fields of culture) with cultural and creative industries – with the word “sectors” denoting both. In 2022, the collective term “cultural and creative sectors” (CCS), or simply “sectors of the creative economy”, was accepted by the expert authorities of the European Commission and UNESCO.

In the present paper, we pose the following research questions: how, if at all, does a mix of harsh market economy mechanisms and the politicization of administration and management processes affect the formation of the country’s cultural policies? If yes, how are we then, from the perspective of European researchers, to understand the effects of combining the state’s tight control and responsibility in China for fostering cultural development with market reality and the digital revolution? These questions are even more viable as China exhibits full autonomy in designing its cultural policies, strictly in line with the political program of the Chinese Communist Party (CCP), and as yet no comprehensive overview of the sectorial policy environment in China has been published. ^2^

### Literature review

There is a significant body of research on different aspects of Chinese CCS, both in China and abroad. Most publications concentrate on what in Chinese is called *wenhua chanye* – the cultural industries. Early studies on this subject in English include, most notably, several publications by Michael Keane, between 2000 and 2014 ^3^. Other, more narrowly framed studies include a brief investigation of the Shanghai cultural industries by White and Xu in 2012 ^4^ or a focus on the film industry by Antonios Vlassis in 2015 ^5^. Yi, Throsby and Gao ^6^ in their research published in 2020 provide details on the effects of central policies on cultural industries development between 2008 and 2015. More recently, Park Sang Do has provided interesting data mining research results on Chinese discourse on CCS ^7^. These publications by Yi, Throsby and Gao as well as Park are surprisingly unapologetic in their enthusiastic endorsement of policies and changes in the field in Mainland China. An excellent, and more balanced study of the cultural sector is provided in Wu Kejia’s book *A Modern History of China’s Art Market*. ^8^ A very good study of changes in Chinese intellectual property law issues if offered by Filippo Gilardi *et al*. in 2023 ^9^. Among a very high number of Chinese-language publications ^10^, a good overview of the CSS policies is offered by Ma Kexin ^11^, while Zhao Kaiqiang and Fan Zhou sketch an accurate picture of the principal directions of sectorial development under the 14 ^th^ Five-year Plan ^12^.

While a number of scholars tackle the issue of cultural industries in Mainland China, there is far less research available on overall, governmental policies directed towards the entire CCS. Michael Keane’s 2007 monograph, Ben Garner’s 2015 paper on the UNESCO Convention on Cultural Diversity ^13^, and Hu Huilin’s historical overview of 2019 ^14^ are some of the very few examples of research on the subject available in English. Among Chinese-language publications which in any way tackle the issue of the Chinese CCS general policy and management, one can mention publications by Wen Jiaohui ^15^; Chen Xiyan and Chen Lixu ^16^; or Xiao Bo and Ning Lanyu ^17^, all quoted in this paper. There is even less information available on the way the cultural sector in China is funded and managed at the central level, a fact that we find quite striking. This is coupled with the scarcity of publicly available data on governmental and other official portals devoted to the field of culture in Mainland China, which also includes the National Bureau of Statistics of China. To our knowledge, no attempt at describing the general characteristics of the sector from the point of view of policy management and funding has been published to date. This paper aims at helping to bridge this gap.

### Methods

In the present research, we used mixed methods typical of the paradigms of the humanities and social sciences. This interdisciplinary approach stems from the very concept of cultural policy, in which various disciplines, from cultural studies to political science, management and cultural economics are interconnected. We used a proprietary research method, which is largely based on desk research analyses of primary sources (legal acts, statistics), and secondary sources (literature, reports) in Chinese and in English, supplemented by media content analysis, and consultations with experts ^18^.

The issue of China's cultural policy was tackled using the Compendium of Cultural Policies & Trends ^19^ grid of issues, positively validated by the creation of European country profiles, as well as a modified version of the profiles of selected Asian countries. For the present analysis, the following list of issues from the Compendium grid was selected:

i. cultural policy system;
ii. domestic governance system;
iii. cultural and creative sectors - institutional landscape;
iv. copyright or intellectual property protection;
v. cultural participation and consumption;
vi. financing and public support.

The authors did not attempt to create a full China country profile as featured in the Compendium. Given the complexity of the cultural policy of such a large country as China, only selected issues were taken into account. It was also challenging to reflect, using the Compendium framework, the dynamic nature of the digital cultural market which in recent years has grown in importance in China. Governmental policies, which we only mention briefly, technological and market developments, and the consequences of the COVID-19 pandemic have all contributed to a surge of artistic activity in the digital sphere, and the embrace of digital art by audiences in the country in the last five years. We are well aware of the specificity of the Chinese party-state, with total control exercised by the CCP at all levels of government. We also acknowledge the CCP’s understanding of culture as a tool serving political goals of the party-state. This, however, does not devaluate research of the country’s cultural policies using the framework described above.

### The concept of contemporary cultural policies

Contemporary cultural policy can be understood as an intentional and systematic intervention of central and local governments in the field of culture and its industries. ^20^ The extent of state interventionism varies: it can occur in a limited formula, typical of the neoliberal approach (where sometimes the cultural policy is to not have any policies), or in a social democratic one, which follows the principle of “the more state presence in culture the better”. ^21^ The goals, principles and priorities of cultural policies also differ. Commonly shared current goals of cultural policy are:

i. preservation of national and cultural identity;
ii. assurance of equal access to culture;
iii. promotion of creative output and high-quality cultural goods and services;
iv. diversification of cultural offer that recognizes the variety of social groups. ^22^

The principles of cultural policies are:

i. right of freedom of artistic creation, education, research and use of cultural assets;
ii. decentralization of decision-making processes regarding the organization and financing of cultural activities;
iii. fostering community participation in decision-making processes by organizing expert panels and initiating public discussions regarding possible solutions for key problems;
iv. ensuring the transparency of decision-making processes;
v. applying the principle of subsidiarity: decisions concerning culture are made by those, to whom they pertain. Central authorities should not make decisions concerning local affairs instead of local governments unless they have been specifically authorized to do so. ^23^

There is common agreement on the necessity that under state interventionism, public funding for culture should be directed to organizations and institutions, artists and creators, and projects. Countries should be free to develop cultural policies in their own way, setting their own goals, principles and determining priorities, without uniformization of cultural institutions and organizations, and operational models. ^24^ A good example of that practice is the fact that all EU member states are setting up their own cultural polices, based on the principle of subsidiarity. As is shown in this paper, while China shares the general goals of cultural policies, its understanding of the principles of these policies is quite different, especially in the field of artistic freedom, and decentralization and transparency of the decision-making process.

## Cultural policy system in the PRC

### Historical background

Culture for the CCP is understood predominantly as one of the management tools in internal politics and public diplomacy, very much in line with the famous Mao Zedong’s 1942 Yan’an Conference on Literature and Art speech in which he likened writers and artists, following Lenin, to the cogs and wheels of the revolutionary machine. ^25^ And while China has transformed itself since that time into a modern country, even today, it is difficult to underestimate the role of politics in the Chinese party-state’s approach to culture. In 2014, Xi Jinping produced a very important address at the Forum on Literature and Art (or rather the Work on Literature and Arts Forum) which as Yang and Jiang ^26^ rightly point out: “demonstrated a strong resemblance to the latter [Mao’s Yan’an speech] in both organization and rhetoric”. While Yang and Jiang attempt to show that Xi’s reiteration is primarily: “a vehicle of the country’s cultural soft power” ^27^, it is much more than this. The speech calls the artists “engineers of the souls” who “wave high the banner of core socialist values” and are burdened with the task of “telling people what should be affirmed and praised, and what needs to be opposed and condemned” ^28^. Later party documents and Xi speeches are less explicit as to the expected role of artists in strengthening socialism with Chinese characteristics. However, documents such as *Xi’s Work Report to the 20th Party Congress* of October 25 ^th^, 2022, and most importantly *Xi Jinping`s Thought on Culture* (October 2023) make it clear that in the party-state ideology: “culture is seen as foundational to the Party’s legitimacy and to the cohesion of the state” ^29^. This shows that the understanding of the role of culture in the P.R.C. despite dynamic changes of the country since the beginning of the ‘Reform and opening up’ period in 1978 has in principle remained in line not only with Mao-era ideology, but more broadly speaking with Leninist principles from the Soviet Union. It is in the USSR, and the whole Communist bloc that culture was perceived as a tool of propaganda and governance, with artists expected to fulfil their political role of motivating the populace to embrace socialist values, and popularising the achievements and benefits of the system. Cultural production was supposed to reflect the tastes, needs and expectations of the masses, as interpreted by the communist rulers. Social realism as the dominant style in literature, art and architecture, was the apotheosis of this theoretical and political framework.

What makes the Chinese cultural model very different from its Soviet benchmark is the early adoption of the economic component in its policy-making and management. According to Hu Huilin, ^30^ cultural policies of the PRC since its establishment in 1949 went through four stages. Until 1956, all private cultural enterprises were nationalized, and what Hu calls the ‘New Democratic’ model of cultural management was terminated. Between 1957–1978 socialist cultural policies with an ideological focus on class struggle were implemented, and the period was marred by the 1966–1976 ‘Cultural Revolution’. The years 1978–2000 mark the market economy transition period, while the years starting from China’s accession to the World Trade Organization in 2001 are the times of the full establishment of market economy policies. ^31^ Hu’s economically focused classification can be supplemented with a culturally focused perspective. This calls for the inclusion in the historical classification of the stage of close copying of the Soviet cultural management system by China until the Sino-Soviet split of 1960, the period of indigenous adaptations within the closed, ideologically-driven system of total state control between 1960–1978, a decade of unprecedented policy liberalization from 1979 to 1989, and the years 1990–2012 as a time of commercialization and marketisation of culture, bold investments in infrastructure, and impressive internationalization, as part of the “Going Global” ( *zouchuqu*) strategy initiated in 1999, and effectuated during Hu Jintao’s term in office (2002–2012). Since the 1990s, and especially following Deng Xiaoping’s famous Southern Tour in 1992, the P.R.C. embraced the path to gradual, free-market economy transformation of the cultural sector. This was caused not only by the general direction of economic reforms in China, but also by the fear that the country’s opening up to global markets and later its WTO accession would cause full domination of the sector by Western influences. Public cultural institutions of the P.R.C. were forced to become industries (the marketisation process – *chanyehua*), so as to make them competitive and more resilient in contact with Western cultural industries. And so the driving force behind the marketisation of the sector was not only an ambition for economic efficiency but also cultural protectionism and concerns of political nature. And so since the 1990s, state-run cultural organizations have been pushed to generate income from ticket sales, establish their own artist management companies, provide services to corporate customers, introduce merchandise, and capitalize on the high-performance fees of the artistic groups they were running. This orientation is observed also in media reforms of the period which, according to a 2016 study by Guosong Shao *et al*. ^32^ were both economic and political in nature: reducing the burden of the sector on the government, incentivising free market economy developments, pushing for profits, and at the same time maintaining political control of the content. This market orientation of the CCS has been coupled with a strong conviction among the CCP decision-makers that culture, in line with Leninist ideology, is an important tool of social education and political power, as well as a source of social cohesion and stability. ^33^ The use of culture for political and economic purposes was underpinned by major reforms in China’s cultural policy. ^34^ The Xi Jinping era (2012 onwards) has been a time of continued market related and modernization efforts, coupled with an increasing role of party-state ideology. An ever-tighter grip of the state on the sector aims at the ideological alignment of cultural contents with the official party line, while a gradual restriction of international, mainly Western cultural influences in China ^35^ is paired with a drive to promote Chinese nationalistic narratives home and abroad.

### Cultural policies: goals and principles

In the PRC, the contemporary understanding of cultural policy includes the following principal areas: protection of cultural heritage, development of public institutions devoted to the cultural sector, development of various cultural fields, organization and development of cultural productions, cultural diplomacy, research on culture, development of the cultural industries, and other tasks, addressing the foreign and domestic policy goals of the PRC. ^36^ The cultural fields included in the cultural policy are performing arts, music, visual arts, cultural and creative industries, folk arts, and national heritage (including intangible heritage). According to Wen Jiaohui, the principal areas of interest in cultural policy include: ‘socialist market economy, political ideology, and the system of public services’. ^37^ To this, one could add that ‘political ideology’ includes both moral education of Chinese citizens at home and public diplomacy abroad.

The concepts behind the cultural policy system in the PRC seem to be an amalgamate of a planned economy based on single-party rule and a strong market-oriented approach, in which state-owned entities compete with private enterprises, and which allows for the survival of those creators, performers and products which can economically sustain themselves without much help from the state. It is important to stress the duality of the Chinese system. This dual reality results in a constant push and pull between the ways and means of top-down governance with Chinese characteristics, and market economy entrepreneurial policies and approaches. ^38^

**Figure 1.**
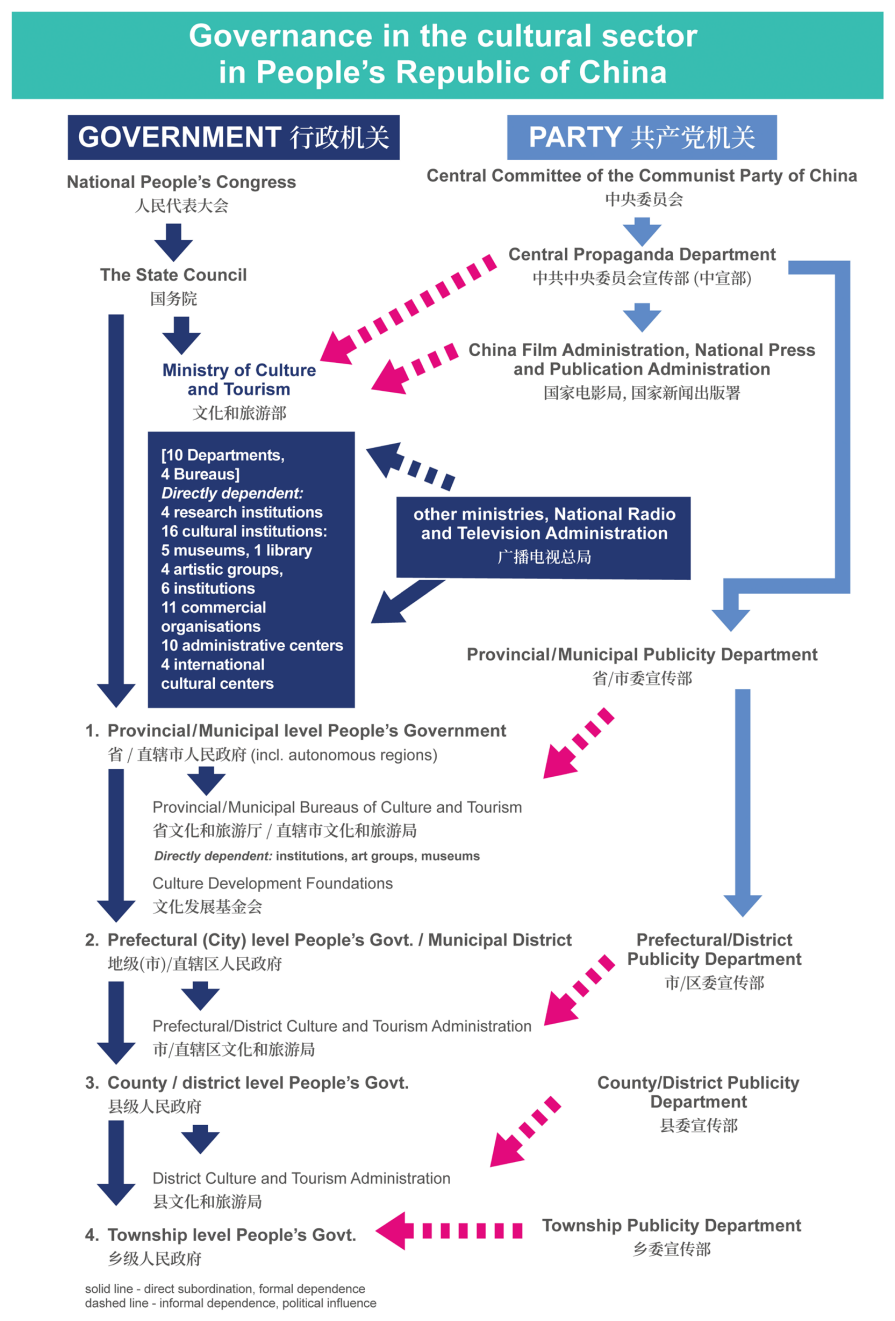
Organigram of the Governance in the cultural sector in the PRC. The Organigram shows in graphic form the organisation of governance of the cultural sector within the Executive branch (labelled “Government”) and the direct subordination dependencies within the Executive on the central and at local levels. It also shows the structure of the Central Propaganda Department branches within the Communist Party of China (labelled “Party”) and their political influence on the Executive. Formal dependencies are marked in solid, blue arrows. Informal dependencies and political influence of the Party on the Executive are marked in dashed, red arrows.

The principles of the formation and implementation of China's cultural policy stem from the broader context of the functioning of the country's state mechanisms. The CCP has the capacity to override most executive structures and procedures. It can enter and regulate the system at any point, either from the very top (the Central Propaganda Department) or through the local Publicity Departments, which are required to approve cultural policies, projects and events from the ideological perspective at their corresponding levels (see Figure 1). The CCP provides general guidelines, leaving the specific administrative policies to the local authorities, while also incentivizing the private sector (including state-owned enterprises). ^39^ Owing to the fact that all decision-makers in the Executive are CCP members and the CCP has the power to interfere at any level, there is no discrepancy between the Party line and the policies enforced by local authorities. While there is the central-local tension (or “experimentation under hierarchy”, as described by Sebastian Heilmann) ^40^, there is no Party-non-Party tension, as no “non-Party” actors exist in the decision-making system. Ministries often jointly propose bills, and various issues are typically tackled by different configurations of ministries with CCP committees, working groups, and other bodies. ^41^

The legal bases of China’s cultural policy are set at the two top levels of strategic planning (national and ministerial) and expressed through two documents:

(1) “Five-year plans” (presently: 14 ^th^ Five-year plan for 2021–2025) by the National Development and Reform Commission, endorsed by the National People’s Congress, formally known as the Plan for National Economic and Social Development ( *Guomin jingji he shehui fazhan guihua*) ^42^

(2) Cultural Development Plans ( *Wenhua fazhan guihua*) – presently, the Cultural Development Plan of the 14 ^th^ Five-year plan, published in August 2022. ^43^

The Cultural Development Plan relates closely to the corresponding Five-year plan, showing the importance of culture in achieving strategic goals of the Five-year plan. All provincial, municipal and other local policy documents are expected to stem from and relate to these guidelines.

According to the latest Cultural Development Plan, adopted in 2022, there are five main, official objectives of the cultural policy in the PRC which could be paraphrased as:

i) increasing cultural and ideological confidence of the Chinese people, as well as their motivation and proactive attitude towards further development of China;

ii) increasing the level of cultural refinement of society, understood as proper behaviour, adhering to moral standards, and exhibiting proper ideological attitudes;

iii) developing the cultural sector as far as the cultural offer to citizens are concerned in cities and the countryside, increasing heritage protection, further integrating culture and tourism, etc.

iv) increasing the international outreach and influence of Chinese culture;

v) continuing the regulation of the cultural sector through the introduction of new laws, and through organizational reforms; increasing overall efficiency. ^44^

An important direction of changes, especially since the outbreak of the COVID-19 pandemic has been on digitalization of the CCS. While culture and digital technology integration was already mentioned in the 12 ^th^ Five-year Plan (2011–2015), later strategic documents only strengthen the central and local government’s resolve to focus on empowering the development of the CCS through new digital technologies. ^45^

An essential principle in the implementation of China's cultural policies is the recognition of censorship as an integral part of the production and presentation process of cultural projects in China. All word and visual content of a given cultural production is screened to determine if the production conforms with the official guidelines before it is allowed to be made public or disseminated. ^46^ Censorship focusses on social norms (restriction of nakedness, sexual content, vulgarity, anti-social behaviour, etc.) and political issues. This holds true both for domestic cultural activity and for those productions from abroad which are allowed to be presented in China. Censorship control is performed by designated bodies in governmental institutions, usually within the Municipal/Provincial Bureaus or at the National Radio and Television Administration, as part of the event acceptation process (the so-called *shenpi*), on the basis of existing legislation such as Regulations on the Administration of Movies (2002), Regulations on the Administration of Commercial Performances (1997), Regulations on the Administration of Audiovisual Products (2002), ^47^ to name a few. No public cultural event can be legally organized without going successfully through *shenpi*, with the exception of events carried out by and on the premises of officially recognized Culture Centres of foreign countries operating in China, such as the Goethe Institute or the Danish Cultural Centre. In the *shenpi* process, financial contracts with artists, details of presentations (times, dates, audiences, etc.), verbal and visual content, as well as legal documentation of the event organizer are examined. The organiser is also required to sign a statement pledging strict adherence to the event plan and to general regulations. Chinese artists, producers and cultural managers usually practice self-censorship in order to avoid having their productions cancelled, removed or in other ways prevented from reaching the audience. As the interpretation of the rules is flexible and exhibits strong regional variations (Beijing as the centre of political power is usually the most restrictive), there is some room for manoeuvre on the fringes of what is allowed. At the same time, approval can be withdrawn without notice, which adds to the uncertainty of the sector (in the past, numerous rock concerts and other popular events were cancelled at the last minute because of such decision shifts).

### Intangible heritage – exemplary priority

On December 2, 2004, China became a signatory to the UNESCO Convention on Intangible Cultural Heritage, and in 2006, the China Centre for the Protection of Intangible Cultural Heritage was established and became the main body responsible for matters related to the protection and development of China’s intangible culture. The tasks of the China National Centre are also carried out at the local level by specially established institutions, responsible for implementing protection measures in specific communities.

From 2017 to 2021, China served its fourth term as a member of the World Heritage Committee since its accession to the Convention. During this latest term, China has adopted a more active attitude in its engagement in world heritage affairs and towards reform of World Heritage mechanisms. Two lines of action in this area are particularly evident. Firstly, the organization of the Youth Forum on Creativity and Heritage, an attempt to integrate the issue of creativity with the preservation of cultural heritage, and secondly, the establishment of the China - Africa Cooperation Initiative for World Heritage, which aims for China to play a guiding role for African countries in the creation and implementation of heritage regulations.

At the same time, increasing globalization and the dynamics of introducing new technologies, as well as China's buoyant economic development, have been contributing to the disappearance of the country's traditional culture not only of ethnic minorities, but also the Han majority, despite all the countermeasures being implemented. ^48^ As early as 2013, the Chinese government has been promoting the convergence of culture with new technologies, expecting that Chinese culture will benefit from the use of new media, in large part by transforming traditional culture into commercialized forms (such as attempts to digitize traditional Chinese calligraphy or a push towards utilizing the NFT ^49^ and the NFT trading market, e.g. by the Poly Auction). This trend is seen more and more each year in legislation favouring the implementation of projects combining art and science or heritage and virtual reality. ^50^

Another dimension of finding a role for intangible heritage in modern CCS is tourism. China identified tourism as a promising part of the CCS early on in the “Reform and opening up” period (1978–2012). While richer cities invested in so-called highbrow culture infrastructure and content, less-developed areas of the country with smaller potential for building audiences for more sophisticated cultural events were incentivised to strengthen their local economies through tourism. Intangible heritage, especially that of ethnic minorities was viewed as a way to draw in domestic tourists from metropolitan areas, looking for new and exotic experiences. Ethnically-focused tourism became a powerful driving force for many local economies, such as Yunnan Province’s Dongba villages, Guizhou Province’s Miao villages or specific, recognisable locations (Yunnan Shangri-La City, Hunan Phoenix Ancient City), etc., creating jobs and generating revenue. Chinese authorities have thus incorporated tourism into their understanding of the CCS, a process symbolically reinforced by the integration of culture and tourism under one Ministry, the Ministry of Culture and Tourism (MOCT) in 2018.

## Domestic governance system

Governance of the cultural sector in the People’s Republic of China can be viewed as a complex interplay between the executive branch institutions and the organs of the Communist Party of China. This decision-making structure is shown in a simplified graphic form in Figure 1. *Organigram of the Governance in the cultural sector in the PRC.*

### National authorities

Major laws regulating the cultural sector are enacted mostly at the central level: this was the case in 19 out of a total of 27 such laws put into force between 2014–2021. ^51^ The main administrative body producing the majority of specific policy documents is the MOCT, but many national guidelines originate at other institutions, sometimes created in collaboration with the MOCT, and sometimes independently. These include the powerful Central Propaganda Department of the CCP, the National Development and Reform Commission, the Ministry of Commerce (especially CCS-connected legislation), the National Radio and Television Administration, and many others. However, there is considerable freedom at local levels to reinterpret the laws, guidelines and regulations produced at the central level and introduce their own initiatives.

There have been many recent changes to the system. In 2018, as part of a major governmental reshuffle, the all-powerful State Administration of Radio, Film and Television (SARFT, *Guojia xinwen chuban guangdian zongju*) was downsized and renamed the National Radio and Television Administration (NRTA, *Guojia guangbo dianshi zongju*), and all responsibilities other than the supervision of the radio and the national television were moved directly to the Propaganda Department of the CCP. As already mentioned, also, in 2018 the Ministry of Culture was renamed the Ministry of Culture and Tourism (MOCT), and the scope of its activities was thus enlarged. The Confucius Institute system (under the Ministry of Education) in 2020 was also reconfigured, with its leading institution, the Office for Chinese Language Council International ( *Hanban - Guojia hanyu guoji tuiguang lingdao xiaozu bangongshi*) downsized and renamed the Centre for Language Education and Cooperation ( *Zhongwai yuyan jiaoliu hezuo zhongxin*), and the modus operandi of the entire system changed.

Considerable stress is put on the development of the CCS, therefore within the MOCT there are not only separate Departments responsible for cooperation with the market, but also four MOCT direct subsidiaries. These are large-scale and influential state-owned companies operating in the CCS market: the China Culture Media Group Co. Ltd., the China Oriental Performing Arts Group Co. Ltd., the China Digital Culture Group Co. Ltd., and the China Animation Group Co. Ltd. Each of these four companies has numerous other subsidiaries. ^52^

### Local authorities

The local level includes provinces and municipalities; prefectures, municipality districts and cities; counties; and townships. The Chinese administrative system at the local level replicates the national system, with four major players: the local CCP Committees, the People’s Congresses (elected, legislative bodies), the People’s Governments (at Provincial/Municipal and lower levels – the main executive body), and the local Committees of the Political Consultative Conference. And so, while the local legislation may come from the CCP Committee or the local People’s Congress, the main institutions responsible for the implementation of national cultural policies at local levels are the People’s Governments. They act through the Provincial Bureaus of Culture and Tourism ( *Sheng wenhua he lüyou ting*), or the Municipal Bureaus of Culture and Tourism ( *Zhixiashi wenhua he lüyou ju*) which are separate administrative bodies (public institutions), directly responsible to the People’s Governments, with an annual budget allocation from the People’s Governments. They also receive funds directly from the MOCT, usually on targeted subsidy programs or specific projects. The Bureaus typically have numerous subsidiaries, e.g. in Beijing there are 13 such institutions (including a library, orchestras, and theatres), and administrative centres, which are separate administrative entities devoted to a certain sphere of activity under the responsibility of the Bureau, for example: Promotion Centre, Cultural Exchange Centre, etc. The Bureaus also actively engage with private or commercial state-run actors, such as the SOEs which run theatre and concert halls belonging to local authorities (e.g. the Poly Group) or organize large-scale festivals (e.g. the China Arts and Entertainment Group).

Districts in major cities, as well as prefectures and townships have some influence on the specific ways policies are implemented locally, but their financial means are limited. They operate local Bureaus of Culture or Cultural Centres, with activities mainly meeting the needs of smaller, local communities. Some bigger and more affluent districts organize larger cultural events (e.g. Chaoyang district in Beijing), but most large-scale support and policy implementation is concentrated at the provincial or municipal level.

### Non-governmental actors

According to Chinese law, private non-profit organizations (NPOs) can be established either as social associations ( *shehui jituan*), social service organizations ( *shehui fuwu jigou*) or foundations ( *jijinhui*) ^53^. All need to be approved by the local Civil Affairs Bureau ( *Minzhengju*). In general, small NPOs operating on a local scale are common, while larger, private not-for-profits active in the field of culture are rare.

There is another type of a “non-profit organization” operating under Chinese law - public institutions ( *shiye danwei*). This is the legal status of most public cultural entities, such as museums, theatres or concert halls. While defined as not-for-profit organizations, they are nevertheless allowed, with the local government approval, to invest and create commercial entities (e.g., subsidiary companies). Profits thus generated - according to the principle of non-distribution constraint - must be redistributed to the statutory purposes of the public institution in question.

A player on the scene enjoying special status is the China Federation of Literary and Art Circles (CFLAC, *Zhongguo wenxue yishujie lianhehui*). It is composed of field-specific associations, such as the China Artists Association, the China Writers Association, the China Film Association, the China Television Artists Association, the Chinese Musicians Association, the China Theatre Association, the China Photographers Association, etc. These associations have branches at provincial and municipal levels, and while they do not have big budgetary means, they still exert a strong influence on artists and creators in their given fields, especially through their personal ties and the institutional leverage of their members. The Federation is a member of the Political Consultative Conference.

## Cultural and Creative Sectors (CCS) – Institutional Landscape

In China’s cultural sector we can distinguish two types of activities:

i) cultural services provided by public institutions, and;

ii) cultural and creative industries (state-owned and private).

Together, they form a part of the creative economy – the CCS.

Cultural services provided by public institutions are not-for-profit oriented, they are publicly funded, non-competitive and occasionally available to the public free of charge (such as museum entries or community artistic performances), especially in smaller cities and towns. These services are designed to meet the basic cultural needs of citizens. Institutions operating in this area are primarily television, radio, public libraries, state museums, art galleries, cultural centres, and in a limited capacity also theatres, concert halls, opera houses and other venues.

Public institutions in the field of culture, although they receive basic operational budgets, are strongly incentivized to generate profit through various activities: ticket sales (season programs and festivals), private sponsorship, venue rentals, commercial events, publications, gifts and souvenirs, etc. To this end they typically establish separately registered, various commercial entities functioning as their commercial subsidiaries. Profits thus generated are redistributed towards the statutory goals of the cultural organizations. All cultural organizations (especially venues) are under this financial pressure to perform economically, including even the MOCT-run or co-run institutions, such as the National Museum of China (NMC), or the National Center for the Performing Arts (NCPA). At the same time, the central authorities initiate various non-commercial programs aimed at increasing citizen participation in culture. These programs include free distribution of tickets for selected performances to local communities, free performances by state-run artistic groups and the like. These programs very often have a negative influence on the market and run against local policies and the commercial performance needs of the venues. Therefore there is strong tension between the pressure to generate profits at the local level, and the public mission of the central authorities. ^54^

Cultural and creative industries are profit-driven and competitive (regardless of whether they are state-owned or fully private). They include publications, certain performing arts, music, film, video and photography, broadcasting, visual arts and crafts, advertising, design and fashion, interactive media and web content, and games. ^55^ Central and local governments have introduced a number of forms of financial support (tax incentives, subsidies, low-interest rate loans) to promote the development of cultural industries. The central government has also initiated major projects such as cultural clusters for specific industries, supporting leading companies and strategic investors, and promoting investment in high-tech cultural assets. ^56^ Local governments have followed these policies with numerous initiatives of establishing cultural innovation industry clusters ( *wenhua chuangyi chanye yuanqu*), aiming at fostering close cooperation between technological service providers and content creators in the CCS. Such creative industries clusters are operating in many major cities, such as the OCT Loft Creative Culture Park (Huaqiaocheng) in Shenzhen or the M50 Creative Space (50 Moganshan Road) in Shanghai, which in recent years has become less of a contemporary art gallery hub, and more of a location for creative content companies. Beijing’s Chaoyang District alone boasts no less than 59 cultural industry parks ^57^ differing in scale and focus. Some real-estate developers, such as Shanghai DOBE Cultural & Creative Industry Development or Shang 8 Culture Group in Beijing specialise in building and running such clusters.

With the push for market reforms of the cultural sector in the last thirty years, much of the market has been taken over by big companies, most of which are state-owned. Poly Group has by far been the most powerful player on the cultural scene for years, with a country-wide network of theatres and concert halls, artistic agencies, productions agencies, touring agencies, one of China’s biggest art auction houses (Poly Auction), an impressive art collection, etc. Originally, Poly Group’s main field of activity had been the military sector (international sales of weapons), from which it moved to the real estate sector, and culture. Other state-owned actors include the Beijing Gehua Cultural Development Group and the China Arts and Entertainment Group (CAEG) – the commercial arm of the MOCT.

The division between public and private actors in the field of culture is very much blurred. Therefore, in Table 1 below showing the number of different types of cultural organizations, we do not take their ownership nature under consideration.

**Table 1.** Public and private cultural organizations by sector in 2021. ^59^

Domain	Cultural institution	Number (2021)	Trend (last 3 years)
Cultural heritage	Cultural relics reservation institutions	2257	-35%
Museums	Museum institutions	5772	+15%
Archives	National Comprehensive archives	3320	flat
Visual arts	Art galleries	682	+c. 15%
Performing Arts	Theatres (general performance venues)	2335	+c. 15%
	Concert houses, music halls	758	+c. 5%
	Performing groups	18370	+c. 5%
Audio-visual	Cinemas	14235	no comparable data
	Radio and TV channels	6554	no comparable data
Interdisciplinary	Cultural centres	43531	flat
Literature	Libraries	3215	flat
	Publishing houses (2020)	586	flat

The infrastructure understood as a venue base is in general excellent. The last 20 years saw massive investments in cultural infrastructure at all levels, with hundreds of new venues (museums, libraries, theatres, concert halls, multi-purpose cultural centres, etc.) being built across the country. Investments in buildings and equipment have not been coupled with investments in human capital – many of the venues have been affected by professional staff shortages, and the equipment is often not used to its full capacity (especially lighting and sound equipment). There have also been many challenges in filling the venues with content – project budgets have been too small, the profit-generation pressure too big, and audience-building is lagging behind especially in second and third-tier cities. The COVID-19 pandemic restrictions, in force until the end of 2022, influenced these processes to a degree that has not yet been assessed, an influence that certainly deserves a separate study.

There are significant differences between the three tiers, into which cities in China are unofficially classified. First-tier cities ( *yixian chengshi*) - Beijing, Shanghai, Guangzhou, Shenzhen - typically have excellent infrastructure, mature audiences, leading festivals, and world-class artists performing there on a regular basis ^58^. The cultural calendars are saturated with events, competition for audiences among venues, and managers is fierce, and the market is very difficult and volatile. Second-tier cities (such as Tianjin, Wuhan, Chengdu, Hangzhou, Ningbo, etc.) typically have good infrastructure, but the audiences are not very mature, and the degree of exposure to world-class culture varies between cities and venues. Third-tier cities are places where international cultural projects are rarely seen, and the quality of the infrastructure base varies from good to very modest. There are some stunning, modern venues, and there are some reputable cultural festivals, but in general, these are the cultural backwaters of China. This division runs parallel with the East-West and the city-countryside income discrepancies. Rich, big cities of the East are contrasted with the poor countryside of central and Western China.

Due to the COVID-19 pandemic and nearly three years of partial or total lockdowns enforced through the zero-COVID-19 policies from January 2020 to December 2022, digital culture initiatives have been on a sharp rise. But the pandemic has only sped up the changes already happening with the advent of a platform economy. China is world No. 1 in e-commerce, and culture is one of many areas in which products are not only sold through Internet platforms but much of the content exists only in digital form. The streaming market in China experienced significant growth during the pandemic, with more than 660 million users utilizing streaming, live-streaming, short film platforms, and apps in 2022. This growth was seen across all age groups and in both first tier and lower-tier cities. The market is projected to maintain a monthly growth rate of approximately 5% in 2022. ^60^

It's important to note that Western video platforms such as Netflix do not operate in China. Instead, the domestic market is dominated by three major online video platforms: iQiyi, Youku, and Tencent Video, which are controlled by the "big three" Internet companies in the country: Baidu, Alibaba, and Tencent (the so-called BAT). Other competitors, such as ByteDance (the parent company of Douyin and its international version - TikTok) have also made great advances on the market. Live streaming platforms are a key sector, with 464 million users and a value of nearly $34 million in 2022. ^61^

The gaming industry is an important part of the digital CCS. Its domestic revenue in 2024 was estimated at almost 40 billion EUR, which makes China the world’s largest gaming market. ^62^ But not only computer games industry and video creators and streaming services embraced the new technological era. Visual artists have used digital tools to find new means of expression ^63^ and communication with their audiences. Virtual and augmented reality, AI content generation, audience participation and co-creation methods are now commonly encountered in works at dozens of art exhibitions across China. Artists and curators broadly use social media channels such as WeChat Moments, Douyin, RedNote (Xiaohongshu) or BiliBili to promote their projects and build connections with their audiences. Cultural content on social media platforms is on a rapid rise. Public institutions are also trying to keep up with the pace of change: museums digitalize their collections, libraries offer new digital services, cultural institutions try to provide new content online. ^64^ This is clearly a visible, long-term trend.

## Copyright or intellectual property protection

### General assumptions

China is a member of the World Intellectual Property Organization (WIPO) and a party to the Berne Convention for the Protection of Literary and Artistic Works. Copyright law in the country is governed by the Copyright Law of the People’s Republic of China, adopted and promulgated in 1990 and revised in 2001. The law protects nine categories of works in China:

i. written works;
ii. oral works;
iii. musical, dramatic, folk, dance and acrobatic works;
iv. works of fine art and architecture;
v. photographic works;
vi. audio-visual works;
vii. graphic and model work, such as engineering design drawings, product design drawings, maps, schematics, etc.;
viii. computer software;
ix. other intellectual achievements that meet the characteristics of a work.

Copyright registration is currently handled by the Copyright Protection Centre of China (CCPC, *Zhongguo banquan baohu zhongxin*), making the system comparable to the US model, where the US Copyright Office plays a similar role. Registration of rights is not mandatory, but is considered proof of copyright ownership, as well as confirmation of the time of the work’s creation.

### Collective Management

The activities of collective management organizations (CMOs) are supervised by the relevant state copyright department. The most important CMOs for collective licensing are:

i. The Music Copyright Society of China (music);
ii. The China Audio-Video Copyright Association (audio-visual works);
iii. The China Written Works Copyright Society (written works);
iv. The China Photo Copyright Association (photographic works);
v. The China Film Copyright Association (films).

According to the report by the International Confederation of Societies of Authors and Composers ^65^ the value of all royalties collected in 2021 was 54.3 million EUR, which ranks China merely 23rd in the world (by comparison, much smaller Poland ranks 19th with a collection of royalties almost twice as large as China’s – 96.7 million EUR). The issue of sending collected royalties that are due to creators from other countries remains unclear. The Music Copyright Society of China declares that 80% of music consumption in China is in Chinese, 10% is by creators coming from Korea and Japan and another 10% from the rest of the world. According to data presented in the organization’s annual reports, about 15% of revenues are shipped overseas, 5% short of the declared collection. ^66^

### Unauthorized use of content

A major challenge to copyright compliance, in China as in other countries is the high level of unauthorized use of cultural content. In its 2021 *Special 301 Report on Copyright Protection and Enforcement*, the U.S.- based International Intellectual Property Alliance (IIPA), pointed out that in the case of China, legislative shortcomings, persistent and growing practices of unauthorized use hindered or completely blocked the ability of rights holders to distribute copyrighted content and prevented rights holders from seeing their investments reach their full potential. ^67^ Contrary to the findings of that report, a study by Giraldi *et al*. 2023 has shown dynamic, positive changes in the way IP protection is viewed and treated in China. IP courts have been opened in Shanghai, Beijing and Guangzhou, handling ever-increasing numbers of IP rights registration applications, and civil and administrative IP violation cases, most of them internet-related. Chinese internet platforms and new media companies generate substantial income from copyright, and so they are instrumental in these changes. As the authors conclude: [in China] *there has been a significant move away from a copycat model to one in which the protection of copyright is ever more important in the Chinese creative industries, a trend which should be viewed within the context of ... China’s obligations as a full member of the WTO*. ^68^

Further development of digital technology and the Internet, big data, artificial intelligence and other cutting-edge technologies will continue to have a far-reaching impact on Chinese society and copyright law. In recent years, copyright issues surrounding artificial intelligence and NFT infringement have brought challenges to existing legal frameworks around the world. China is at the forefront of the process of adjusting the legal system to fit these new challenges and developments, and effective IP protection is in the interest of domestic new media companies and streaming services.

## Cultural participation and consumption

The National Bureau of Statistics of the PRC does not provide any data on household expenditure on culture. Available data shows only expenditure on culture, education and leisure as a single figure, with no further breakdown. A national survey on cultural participation has not yet been conducted in China. Nevertheless, in 2015, a partial survey was conducted by the National Institute for Cultural Development at Wuhan University, with support from the Ministry of Culture, in 13 different Chinese cities (all were important political, economic and cultural centres representing different regions of China) ^69^. The survey used a face-to-face interview method, however, the sample was not representative of the Chinese population, which makes the study different from most national surveys of cultural participation. Courty and Zhang ^70^ undertook an analysis of the results of the Wuhan University study and pointed out that education is crucial in increasing cultural participation. Income has a smaller but noticeable impact on the frequency of cultural consumption. In their assessment, strong evidence can be found in support of the elitism hypothesis for the so-called highbrow culture (library, museum, gallery and art performance). This suggests that cultural participation in China fits the patterns observed in high-income countries. There are essential differences in cultural participation across surveyed cities for all cultural indicators. For all non-public indicators excluding art performance, city differences do not appear to be correlated with income. For highbrow culture (art performance and public cultural activities) however, the plots display a U-shape dependence on development in relation to urban growth. Middle-income cities tend to have lower levels of cultural participation. This is true for highbrow culture, and also when one latent variable affects all indicators of cultural participation. The impact of education, and to some extent income, is weaker in wealthier cities whereas data on cultural participation in rural areas is still lacking.

### Trends and figures in household expenditure

From 2005–2013, culture consumption increased in absolute values as well as a fraction of total consumption in both rural and urban areas. At the same time, cultural consumption was significantly higher in urban areas, both in absolute and percentage terms (see Table 2 and Table 3).

**Table 2.** Urban vs rural cultural consumption: 2013–2015 ^71^.

Year	Urban households			Rural households		
	Per capita annual cultural consumption (RMB)	Per capita annual cultural consumption (EUR) *	Share of total consumption (%)	Per capita annual cultural consumption (RMB)	Per capita annual cultural consumption (EUR) *	Share of total consumption (%)
2013	945.7	135,60	5.1%	174.8	25,06	2.3%
2014	1087.9	155,99	5.4%	207.0	29,68	2.4%
2015	1216.1	174,37	5.7%	239.0	34,27	2.6%

*Based on the average RMB – EUR exchange rate in 2015 (1 EUR = 6.9743 RMB)

**Table 3.** Income and Expenditure of Urban and Rural Households in 2021.

ITEM	Absolute Value (RMB)	Nominal Increase Y/Y (%)	Absolute Value (EUR)
**Total Per Capita Expenditure of Urban Households** Grouped by Consumption Category	**30307**	**12.2**	3969,85
Food, tobacco and liquor	8678	10,1	1136,71
Clothing	1843	12,0	241,41
Residence	7405	6.4	969,96
Household facilities, articles and services	1820	11.0	238,40
Transportation and telecommunication	3932	13.2	515,04
Education, culture and recreation	3322	28.2	435,14
Health care and medical services	2521	16.1	330,22
Miscellaneous goods and services	786	21.7	102,96
ITEM	Absolute Value (RMB)	Nominal Increase Y/Y (%)	Absolute Value (EUR)
**Total Per Capita Expenditure of Rural Households** Grouped by Consumption Category	**15916**	**16.1**	**2084,80**
Food, tobacco and liquor	5200	16.1	681,14
Clothing	859	20.6	112,52
Residence	3315	11.9	434,22
Household facilities, articles and services	900	17.3	117,89
Transportation and telecommunication	2132	15.8	279,26
Education, culture and recreation	1645	25.7	215,47
Health care and medical services	1580	11.4	206,96
Miscellaneous goods and services	284	26.5	37,20

*Based on the average RMB – EUR exchange rate in 2021 (1 EUR = 7.6343 RMB)* *Source: National Bureau of Statistics of China*

**Figure 2.**
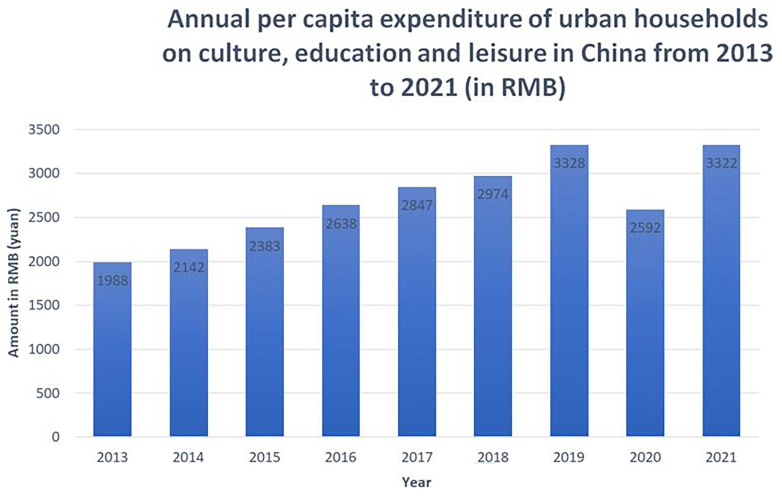
Annual per capita expenditure of urban households on culture, education and leisure in China from 2013 to 2021 (in RMB). The Figure shows gradual increase of per capita expenditure between 2013 and 2019, a drop in 2020 attributed to the COVID-19 pandemic, and a recovery trend in 2021. Source: National Bureau of Statistics of China.

In 2013–2021, the upward trend was maintained, with the exception of 2020 when cultural participation was significantly reduced, due to the pandemic and the zero-COVID policy lockdowns (see Figure 2). It is expected that a similar reduction will be shown for 2022, once the data is available.

**Figure 3.**
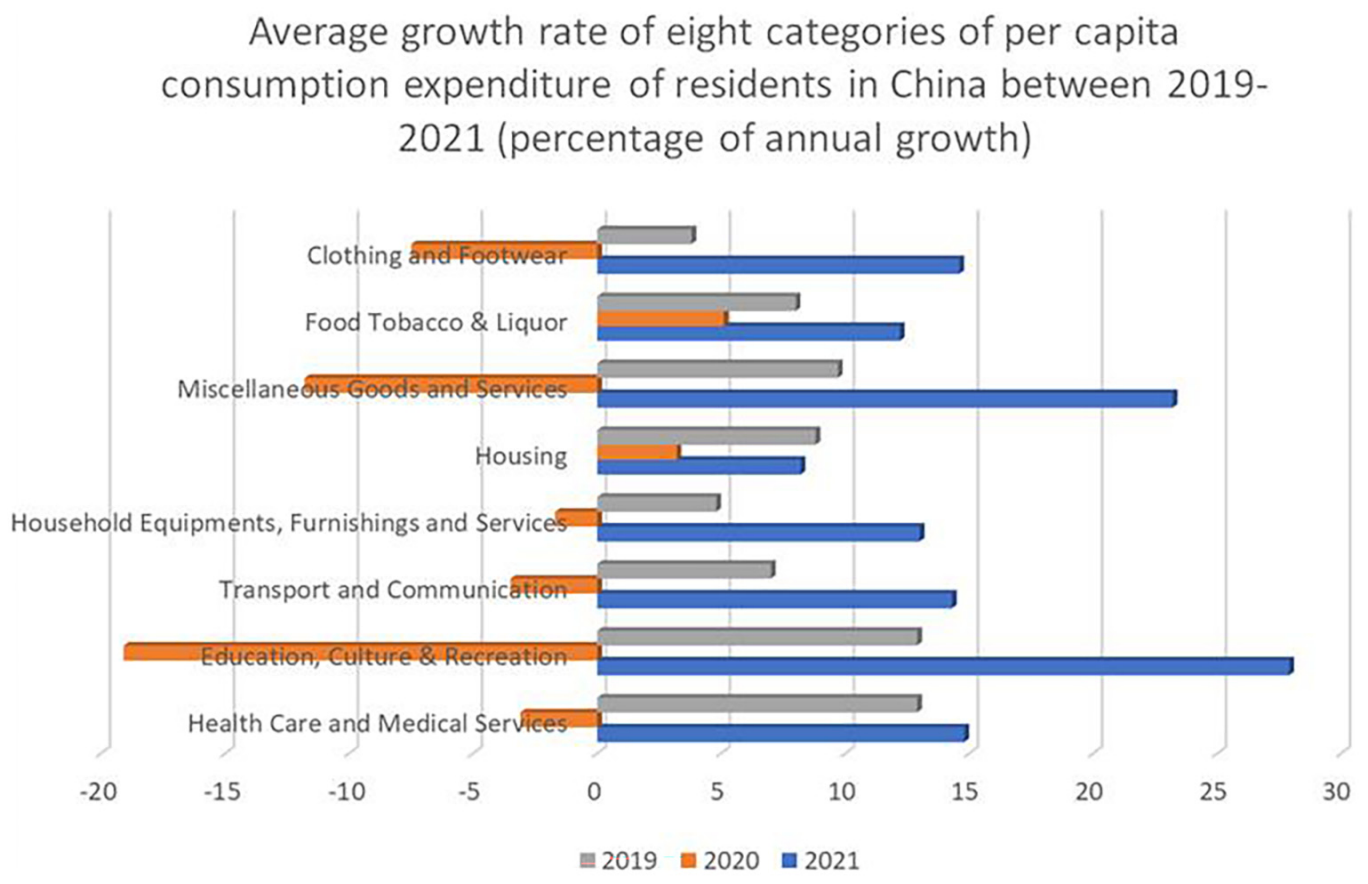
The average growth rate of eight categories of per capita consumption expenditure of residents in China between 2019–2021. The Figure shows the average growth rate of eight categories of per capita consumption expenditure divided between the years 2019, 2020, and 2021. The Figure shows the biggest decrease in consumption in 2020 in the “Education, Culture & Recreation” category (-19,1%), presumably due to the COVID-19 pandemic, and also the biggest recovery in 2021 (+27,9%). Source: National Bureau of Statistics of China

In 2021, as reported by the National Bureau of Statistics of China, compared to 2019, the average growth rate of per capita spending on culture, education and leisure for Chinese residents was 1.7%. As the three types of expenditures are grouped together, it is difficult to estimate what part of these figures represents expenditure on culture. Based on the experience of European countries, it can only be assumed that a significant proportion of this amount is spent on education and leisure, while culture accounts for a much smaller proportion. Figure 3 shows the average growth rate of eight categories of per capita consumption expenditure of residents in China between 2019–2021. Education, culture and recreation data is bound together, and shows a 10,8% growth – significantly higher that the growth in household goods and services, and two percentage points higher than the growth in health care and medical services.

## Financing and public support

Although China has seen strong economic and social development over the past 40 years, growth in the cultural sector is relatively recent. Accordingly, the added value of culture grew at a rate of about 15–20% in the decade, 2004–2014. ^72^ The major indicators for culture are difficult to determine using the National Bureau of Statistics data, as it provides public expenditure in culture combined with sport and media. And so, in 2021, public culture, sport and media expenditure per capita was 282.24 RMB (38.67 EUR calculated at 07.01.2023 mid-market rates). Public culture, sport and media expenditure in percentage of GDP stood at 0.34% and amounted to 1.62% of total public spending.

The amount of public subsidies funded by the central government exhibits strong geographical variation. In the case of museums, for example, which have been built on a massive scale in China in recent years, the level of public support is usually lower in richer Eastern provinces (20%), average in central provinces (60%) and highest in less rich Western provinces (80%). ^73^ Both central and local governments are committed to providing free cultural services through the development of cultural infrastructure.

Local authorities are powerful players in the cultural sector with public spending levels reaching 94% of total spending in 2021 (see Table 4). The metropolitan areas' budgets for culture are unusually high. For example, in 2021 the Beijing municipal budget stood at 75 billion EUR, and the budget for culture, tourism, sport and the media amounted to 1,4 billion EUR (1,87% of the total budget). ^74^ Major municipalities run Culture Development Foundations, which allocate budgets to artistic groups, cultural institutions, and projects. In general, the specific amounts allocated to individual applicants are not disclosed, the exception being, e.g. the Shanghai Culture Development Foundation. ^75^

**Table 4.** Public expenditure on culture at the central and local levels in 2021.

Level of government	Total expenditure in RMB	Total expenditure in EUR *	% share of total
Central	21,113,000,000	2,892,500,000	5,3%
Local	377,410,000,000	51,731,436,199	94,7%
TOTAL	398,523,000,000	54,625,386,578	100%

* *Based on the RMB – EUR mid-market exchange rate as of 2023.01.07 (0.137)* Source: National Bureau of Statistics

### Expenditure per sector

The MOCT budget provided on the official MOCT website has a very general cost breakdown, providing little about specific budget allocations across different categories (see Table 5 below). The MOCT can apply for additional special program budgets to the Ministry of Finance, and the funds thus obtained are redistributed to local authorities at proportions predetermined by the CPC.

**Table 5.** The Ministry of Culture and Tourism 2022 budget.

	Amount in thousand RMB	Amount in thousand EUR *
Administration	202,414.7	27,730.8
Daily administrative management costs	14,246.0	1,951.7
Performing groups	20,256.9	2,775.2
Cultural events	173,605.8	23,783.9
Cultural creativity and protection	37,938.0	5,197.5
Management of CCS and tourism industry	28,029.0	3,839.9
Management of culture and tourism	156,847.8	21,488

* *Based on the RMB – EUR mid-market exchange rate as of 2023.01.07 (0.137)* Source: Ministry of Culture and Tourism ^76^

## Conclusion

The PRC is a country deserving special attention in cultural policy research due to its sheer size, rapid modernization, sui generis political system, and increasing global influence. This paper aims to outline a general picture of the Chinese cultural policy environment, as such a picture seems to be lacking in scholarly publications on China. The outcomes of the research presented here alongside the practical knowledge provided by experts working in China, show several main characteristics. Firstly, the cultural policies of the PRC are a function of the central-level political policymaking of the Chinese party-state. Culture is highly politicised, being understood as one of the tools the CCP used to cement its power and ensure social stability. Secondly, the cultural sector is subject to total control by the party-state through the CCP Propaganda Department, and the censorship system. This not only affects the sector domestically but hinders China from a fuller and successful engagement with the international cultural field. Thirdly, the PRC’s cultural sector exhibits dual characteristics, with market-insulated public services on the one side, and the market economy CCS, where state-owned enterprises compete for services, customers, and profits with private entities on the other. Furthermore, the policy and domestic governance systems show incoherency, exhibiting tensions between central-level general policy guidelines and local-level implementation; the mission of the state to ensure wide cultural participation and market economy incentivization of public cultural organizations. Numerous policies foster robust participation of private enterprises in cultural and creative sectors, while the cultural market is dominated by an intricate network of state-owned players, such as the Poly Group, the Ministry of Culture and Tourism and local authorities’ commercial subsidiaries. Lastly, the legal environment in the PRC is dynamically changing, as shown by the recent introduction of comprehensive IP protection policies. Changes are also visible in the broad digitalization of culture, a trend greatly accelerated during the COVID-19 pandemic.

Due to the scarcity and poor quality of statistical data, it is difficult to fully assess culture expenditure and consumption trends in the PRC. Available data hints at surprisingly modest public expenditure on culture at the central level, and large public expenditure at the provincial and municipal level, cascaded to lower-level local authorities. The overall view of the sector shows a very dynamic and unique system constantly redefining itself to cater for ambitious, and often conflicting goals set by the central authorities.

## Ethics and consent

Ethical approval and consent were not required.

## Data Availability

The statistical data used in this paper is available in Open Access under CC 4.0 license at Zenodo: https://doi.org/10.5281/zenodo.15376612 This project contains the following underlying data: Data/Material file 1. (Central Expenditure Statistics - *National Bureau of Statistics of China*) Data/Material file 2. (General Data of Cultural Institutions - *National Bureau of Statistics of China*) Data/Material file 3. (Local Expenditure - *National Bureau of Statistics of China*) Data/Material file 4. (Museums - *National Bureau of Statistics of China*) Data/Material file 5. (National Expenditure - *National Bureau of Statistics of China*) Data/Material file 6. (Performance Groups - *National Bureau of Statistics of China*) Data/Material file 7. (Education, Culture & Recreation Expenditure of Urban Households (yuan) - *National Bureau of Statistics of China*) Data/Material file 8. (Per Capita Expenditure of rural households 2021- *National Bureau of Statistics of China*) Data/Material file 9. (Per Capita Expenditure of urban households 2021- *National Bureau of Statistics of China*) Data/Material file 10. (The average growth rate of eight categories of per capita consumption expenditure - *National Bureau of Statistics of China*)
