# Supplementary material for: The cultural sector in China through the lens of cultural policies concepts
Source: Open Res Eur. 2025 Oct 22;5:217. Originally published 2025 Aug 4. [Version 2] doi: 10.12688/openreseurope.20127.2 (PMC12576313; doi:10.12688/openreseurope.20127.2)
Supplement: Peer review history

# Reviewer report: Reviewer response for version 2

**DOI:** 10.21956/openreseurope.23281.r62817

**Published:** 2025-11-22

**Authors:** Carole Rosenstein

**Affiliations:** 1 George Mason University, Arlington, USA

This article offers a profile of the cultural policy system in place in China, providing valuable information that is not readily available to cultural policy scholars in other parts of the world. Using the established Compendium of Cultural Policies & Trends template, the authors describe key priorities, components, and characteristics of the Chinese system and map this system in a useful organigram.

 A strength of the article is that, like other profiles produced for the Compendium, its careful design enables detailed and deep description that provides real insight into the case. Particularly in its revised form, the article is notably comprehensive in regard to expressive forms. One place where information remains lacking is the system for arts education and professional certification, and a description of how that system intersects with the cultural bureaucracy lain out here. It would be a great benefit to include that component.

 Another strength of the article is that enables valuable conceptual comparisons between China and other nations that are similarly described in the Compendium. For example, the article maps the high degree of vertical centralization that characterizes the Chinese cultural bureaucracy, while noting its bifurcated decentralization along the horizontal dimension. It is interesting to think about how aspects of culture are hived off in national cultural bureaucracies, and what such structural patterns can tell us about the cultural ideologies at play. As the authors note, this pattern reflects the primacy of classic Marxist ideologies about the role of culture in social and political technologies.

 The authors’ discussion of the ways in which the public priorities of democratizing the arts and culture and monetizing and profiting from cultural activities clash in this case is most interesting. I would have appreciated more analysis of what lessons this might lend to cultural policymakers in other nations.

 I will take advantage of the unusual opportunity this platform affords to “cross talk” with our fellow peer reviewers to say that I disagree with Dr. Jelincic’s suggestion that this piece does not deserve the status of “research article” because it fails to adequately formulate hypotheses or research questions and test them using a defined methodology. In my view, the article engages in research by framing this case using the Compendium template (that is its method), enabling descriptive and comparative analysis such as the examples I mention above.

 While it is important to maintain research standards in our still emerging field, I see over the past few years a disturbing trend of making a fetish of methods. The article is a valuable inquiry into this case, the driving principles that make this system work, and the contradictions it poses when faced with politics IRL. Scholarly inquiry need not be explanatory to be recognized as and earn the honorific of research. If we demand that, we will curtail the strength cultural policy study draws from our interdisciplinarity.

If any, are all the source data and materials underlying the results available?

Partly

Is the work original in terms of material and argument?

Yes

Is the work clearly and cogently presented?

Yes

Is the argument persuasive and supported by evidence?

Yes

Does the research article contribute to the cultural, historical, social understanding of the field?

Yes

Does it sufficiently engage with relevant methodologies and secondary literature on the topic?

Yes

Reviewer Expertise:

Cultural Policy, Arts Policy, Arts Management

I confirm that I have read this submission and believe that I have an appropriate level of expertise to confirm that it is of an acceptable scientific standard.

# Reviewer report: Reviewer response for version 2

**DOI:** 10.21956/openreseurope.23281.r62821

**Published:** 2025-11-06

**Authors:** Chiara Burlina

**Affiliations:** 1 University of Padova, Padua, Italy

Comments to the Authors

 I thank the Editors for the opportunity to read the reviewed version of the manuscript “The cultural sector in China through the lens of cultural policies concepts.”.

 The paper addresses an important topic — the development of cultural policies in China — with a focus on how these policies are perceived domestically and internationally, among practitioners and academia. The topic is relevant to current academic and policy debates. I acknowledge the Authors for the intensive revisions after round 1, the paper has significantly improved with respect to its initial version. Yet, in my opinion, it requires some additional minor adjustments before final publication.

 I would suggest to the Authors to define in the Introduction which are the creative and cultural sectors they are looking at. They report them clearly in Table 1 at p.12, however I think that is more straightforward to have a brief definition at the very beginning of the paper.

 Secondly, the sentences that explore two Research Questions at p.3 are quite too long to be followed. I would rephrase them in a shorter and more immediate manner.

 Third, in the Conclusions, I do appreciate how the Authors highlight the main policy implications for the development of Chinese cultural policies. However, I would add a small paragraph to future research opportunities for example including a comparison between China and the rest of the World. Meaning, how China could improve/change its policy environment with respect to Europe or other developed countries?

If any, are all the source data and materials underlying the results available?

Yes

Is the work original in terms of material and argument?

Yes

Is the work clearly and cogently presented?

Yes

Is the argument persuasive and supported by evidence?

Yes

Does the research article contribute to the cultural, historical, social understanding of the field?

Yes

Does it sufficiently engage with relevant methodologies and secondary literature on the topic?

Yes

Reviewer Expertise:

Cultural and Creative Industries, Institutions, Social Capital

I confirm that I have read this submission and believe that I have an appropriate level of expertise to confirm that it is of an acceptable scientific standard, however I have significant reservations, as outlined above.

# Reviewer report: Reviewer response for version 2

**DOI:** 10.21956/openreseurope.23281.r62822

**Published:** 2025-11-04

**Authors:** Daniela Jelincic

**Affiliations:** 1 Institute for Development and International Relations, Zagreb, Croatia

The article deals with the cultural sector in China through the lens of cultural policies concepts. It mainly does so by focusing on the CCS, which is why the authors may consider putting it into the title. The method applied relies on the desk research (available statistics and descriptions) pertaining to the Compendium structure. While application of this method results with a text which is highly informative for a reader (especially non-Chinese ones), it still remains within the confines of a report. It is clear that preparing such a report was not easy due to a number of justified reasons (e.g. lack of readily available statistics, non-English sources, etc.). However, to be able to differentiate a report from a research article, additional work is required. This primarily entails formulating a valid hypothesis or research questions (RQs). While the authors have formulated RQs, they neither seem to be clearly answered in the text, nor is the methodology clear enough in explaining how the methods applied have helped in replying to those questions.

 Namely, the 2 RQs are the following:

 1. how, if at all, does a mix of harsh market economy mechanisms and the politicization of administration and management processes affect the formation of the country’s cultural policies? (minor suggestion: reformulate the question in a way that it first asks, "Does a mix of..." and then "if, so - how?")

 2. If yes, how are we then, from the perspective of European researchers, to understand the effects of combining the state’s tight control and responsibility in China for fostering cultural development with market reality and the digital revolution?

 Although the RQs are present, we do not learn much about the answers, especially related to the 2nd RQ. It is therefore suggested to provide clear answers to them and to explain how methodology supported the process in revealing the answers to those RQs.

 Further on, if selected chapters from the Compendium grid were used for the analysis (6 of them), the authors should explain why those chapters were used to justify the selection, and how each of the topics selected relates to the RQs.

 As said, the text is highly informative, but some parts seem to be overwhelming and draw the reader's focus away from the main topic (RQs) of the article. E.g., while it is really interesting to read about the historical background of the cultural policy system in China, it is not necessary for performing the focus of the research.

 Similarly, when explaining the aim of the article, the authors are not clear enough claiming the aim being the following:

 1. how the People's Republic of China (PRC) shapes its cultural policies within the framework of contemporary cultural policies theory and international practice

 2. To our knowledge, no attempt at describing the general characteristics of the sector from the point of view of policy management and funding has been published to date. This paper aims at helping to bridge this gap.

 3. This paper aims to outline a general picture of the Chinese cultural policy environment, as such a picture seems to be lacking in scholarly publications on China.

 In 1. it seems that the goal is on detecting the modality of shaping PRC cultural policy/ies within the framework of contemporary cultural policies theory and international practice (not much is said about international practice).

 In 2. and 3. the aim is to offer a general overview of the PRC cultural policy environment (in 2. the focus is on policy management and funding, although other topics are also analyzed, e.g. cultural participation and consumptions). This requires further clarifications.

 Further on, the expressed aim is not totally in line with RQs.

 When it comes to the structure of the article, the authors state that they used methods usually applied in the humanities and social sciences, which may explain the unusual, rather descriptive style and pertaining structure, lacking the sections of Results and Discussion. While it may be acceptable to a point, the suggestion is to either add the Discussion section or reinforce the Conclusion section putting the findings in the relation with the existing theoretical works cited in the introductory parts of the article. This would possibly offer some insights and answer the RQs, especially RQ2.

 Finally, I would like to say that, especially from a European point of view, the article offers extremely valuable data (although incomplete due to the objective reasons), and as such may serve as an important point of entry not only for understanding the PRC cultural policy but also having implications on further research, policy (cultural, economic, foreign...) as well as managerial implications. This is why the authors should understand these suggestions as a motivation for the enhancement of the article since it may have very important implications.

If any, are all the source data and materials underlying the results available?

Yes

Is the work original in terms of material and argument?

Yes

Is the work clearly and cogently presented?

Partly

Is the argument persuasive and supported by evidence?

Partly

Does the research article contribute to the cultural, historical, social understanding of the field?

Yes

Does it sufficiently engage with relevant methodologies and secondary literature on the topic?

Partly

Reviewer Expertise:

Cultural policy, cultural/creative industries, cultural heritage, cultural tourism

I confirm that I have read this submission and believe that I have an appropriate level of expertise to confirm that it is of an acceptable scientific standard, however I have significant reservations, as outlined above.

# Reviewer report: Reviewer response for version 2

**DOI:** 10.21956/openreseurope.23281.r62619

**Published:** 2025-10-30

**Authors:** Michael Keane

**Affiliations:** 1 Queensland University of Technology, Brisbane, Australia

With regard to endnote 29, the report is not refeered to officually as "Xi's report". It should read as "The Report to ....by Xi Jinping on behalf of the 19th CCP Central Committee". The quote in the sentence comes not from Xi but from the author of China Brief, so this could be made clearer 

 With regard to the statement "The National Bureau of Statistics of the PRC does not provide any data on household expenditure on culture', it would be better to rephrase as "does not provide differentiated or itemised household expenditure ..." or words to that effect

If any, are all the source data and materials underlying the results available?

Yes

Is the work original in terms of material and argument?

Yes

Is the work clearly and cogently presented?

Yes

Is the argument persuasive and supported by evidence?

Yes

Does the research article contribute to the cultural, historical, social understanding of the field?

Yes

Does it sufficiently engage with relevant methodologies and secondary literature on the topic?

Partly

Reviewer Expertise:

Cultural and media policy in China

I confirm that I have read this submission and believe that I have an appropriate level of expertise to confirm that it is of an acceptable scientific standard.

# Reviewer report: Reviewer response for version 2

**DOI:** 10.21956/openreseurope.23281.r62618

**Published:** 2025-10-30

**Authors:** Andrew White

**Affiliations:** 1 King’s College London, London, England, UK

No further comments as I am satisfied with the changes

If any, are all the source data and materials underlying the results available?

Yes

Is the work original in terms of material and argument?

Yes

Is the work clearly and cogently presented?

Yes

Is the argument persuasive and supported by evidence?

Yes

Does the research article contribute to the cultural, historical, social understanding of the field?

Yes

Does it sufficiently engage with relevant methodologies and secondary literature on the topic?

Yes

Reviewer Expertise:

Chinese cultural policy; Chinese creative industries

I confirm that I have read this submission and believe that I have an appropriate level of expertise to confirm that it is of an acceptable scientific standard.

# Reviewer report: Reviewer response for version 1

**DOI:** 10.21956/openreseurope.21771.r57869

**Published:** 2025-09-02

**Authors:** Andrew White

**Affiliations:** 1 King’s College London, London, England, UK

This is a well-structured paper whose arguments and working definitions are clearly laid out in the opening sections. The paper refers to an impressive range of academic scholarship on the cultural and creative sectors (hereafter the CCS) in China. While there are disadvantages to presenting a overview of the sectors rather than a study with a narrower focus, the dearth in the existing literature of a comprehensive analysis of this kind justifies this approach. Another original aspect of this paper is its utilization of a number of Chinese language sources. Significantly, this included information given to the authors by those working in the Chinese CCS as well as employees of the People’s Republic of China’s Ministry of Culture and Tourism. Given the difficulty of eliciting candid comments in an increasingly politically restrictive environment, the authors are to be commended for their success in generating meaningful insights from their interlocutors.

 The authors provide a good historical analysis of the development of the Chinese economy since the creation of the People’s Republic of China. This enables them to outline their main argument about Chinese cultural policy: that it is increasingly restrictive with regard to the CCS’s content while being largely hands-off in terms of ownership of the means of production of that content. While I would agree with this general argument, I think that a more detailed analysis of the market reforms in the Chinese media sector in the 1980s and 1990s would have bolstered the authors’ stance (Akhavan-Majid 2004; Hong & Cuthbert 1991).

 The organigram that the authors devised is very effective in giving the reader a sense of how Chinese cultural policy actually operates. This is augmented by a critical analysis of this operational structure. This is an important section in that it highlights the limitations of earlier literature which emphasised the constraints on the national government’s capacity to impose its blueprint throughout the country. That is not to say that literature written around 10-15 years was necessarily wrong at that time; rather, it demonstrates the profound tightening of central government political control in the Xi era. This is something that is often under-played in analyses that over-rely on those older sources and thus illustrates the importance of this paper in updating our understanding of China’s cultural-policy-making process.

 The authors sometimes find official statistics on China’s CCS of limited value in terms of the overall analysis, a methodological point that is often missing in many analyses of the country’s cultural policy. This is often because of the amalgamation of distinctly different activities into one category; an example of this is the grouping together of education, culture and leisure (p. 15) which makes it virtually impossible for researchers to make precise assessments of each sector. The authors might have expanded this into a more philosophical discussion about the place of CCS in the Chinese economy and society. Despite the market reforms from 1978 onwards, early approaches to the development of CCS in many cities and regions of China focused heavily on tourism (Keane 2007), a category that is often missing from CCP mapping documents in other countries. This is partly because the national governments desire to promote the type of intangible heritage outlined in this paper as an antidote to the more politically subversive forms of creativity that are de rigour among many ‘western’ artists (O’Connor and Gu 2006; Keane 2006). As important to this is the Chinese state’s reluctance to produce the kind of national mapping document that is a feature of cultural policy in many other countries. The reason for this is that the country’s diversity, not least in levels of development between the provinces on the eastern seaboard and those in the western interior, renders this a largely futile exercise (White & Xu 2012). This enables the poorer provinces free to focus on intangible cultural heritage and the tourism that goes with it at the expense of the CCS that predominate in the first-tier cities. This is a discussion which the authors might have developed in their own section on intangible cultural heritage and tourism.

 I will finish by focusing on a couple of areas where China has had some success in the development of its CCS. The paper argues that online piracy of CCS content, including from overseas sources, is rampant. However, the main source for this assertion is an International Intellectual Property Alliance (IIPA) (2021) report whose evidence for this is unconvincing. In particular, the lack of comparative figures for other countries is a significant omission. This is important because it under-plays the extent to which piracy still continues to be a problem even in those countries that have put robust technical and legislative systems in place to deal with it. To give one example, a UK House of Commons Select Committee report on music streaming in 2021 highlighted the continuing piracy of songs by UK users. What we do know about China is that significant efforts have been made in recent years to curtail the circulation of pirated CCS content in online spaces. This includes the opening of courts specifically devoted to IP violations in 2014-15 in Shanghai, Beijing and Guangzhou. This built on the increasing assertiveness of Chinese courts in the 2010s in prosecuting not only copyright infringements related to domestic content, but also a considerable number of cases where the plaintiffs were from overseas (Thomas 2017). (A summary of all these developments can be read in a paper that I co-authored in 2022 (Gilardi et al. 2022)). These developments should be considered by the authors in their future research on the Chinese CCS.

 Finally, it is worth noting that the increasing censoriousness of Chinese society has not stopped the country’s CCS professionals from flourishing on the world stage. At the time of writing, Pop Mart’s Labubu dolls are taking the world by storm. Similar to the way in which the censorship regime in Elizabethan England did not prevent Shakespeare and other writers from weaving their magic, we should take heart from the way in which contemporary Chinese creatives too can subvert the dead hand of the censor.

If any, are all the source data and materials underlying the results available?

Yes

Is the work original in terms of material and argument?

Yes

Is the work clearly and cogently presented?

Yes

Is the argument persuasive and supported by evidence?

Yes

Does the research article contribute to the cultural, historical, social understanding of the field?

Yes

Does it sufficiently engage with relevant methodologies and secondary literature on the topic?

Yes

Reviewer Expertise:

Chinese cultural policy; Chinese creative industries

I confirm that I have read this submission and believe that I have an appropriate level of expertise to confirm that it is of an acceptable scientific standard.

## References

[1] : The cultural sector in China through the lens of cultural policies concepts. *Open Research Europe* .2025;5: 10.12688/openreseurope.20127.1 10.12688/openreseurope.20127.1 41181068 PMC12576313

[2] : Mass Media Reform in China. *Gazette (Leiden, Netherlands)* .2004;66(6) : 10.1177/0016549204047576 553-565 10.1177/0016549204047576

[3] : From copycat to copyright: intellectual property amendments and the development of Chinese online video industries. *International Journal of Cultural Policy* .2023;29(2) : 10.1080/10286632.2022.2040494 152-168 10.1080/10286632.2022.2040494

[4] : Media reform in China since 1978: Background factors, problems and future trends. *Gazette (Leiden, Netherlands)* .1991;47(3) : 10.1177/001654929104700301 141-158 10.1177/001654929104700301

[5] : From made in China to created in China. *International Journal of Cultural Studies* .2006;9(3) : 10.1177/1367877906066875 285-296 10.1177/1367877906066875

[6] Keane, M. (2007) Created in China: The great new leap forward. London & New York: Routledge.

[7] : A new modernity?. *International Journal of Cultural Studies* .2006;9(3) : 10.1177/1367877906066874 271-283 10.1177/1367877906066874

[8] Thomas, K. (2017) Assessing intellectual property compliance in contemporary China: the World Trade Organisation TRIPS Agreement. Singapore: Palgrave Macmillan.

[9] : A critique of China's cultural policy and the development of its cultural and creative industries: the case of Shanghai. *Cultural Trends* .2012;21(3) : 10.1080/09548963.2012.698558 249-257 10.1080/09548963.2012.698558

[10] Keane, M. (2007) Created in China: The great new leap forward. London & New York: Routledge.

# Response

**Published:** 2025-09-27

**Authors:** Marcin Jacoby

**Affiliations:** Faculty of Humanities, SWPS University of Social Sciences and Humanities, Warsaw, Masovian Voivodeship, Poland

Dear Professor White, Thank you very much for your very fast and thorough review of our paper and for providing us with so many suggestions on how to improve our text. We have tried to address all of the issues you pointed out, introducing the following changes:

- we have added some information (and a reference to a useful paper) on media reforms
- we have added some new content on the understanding on CCS in the P.R.C., drawing some parallels with the USSR and the Soviet bloc
- we have added a new paragraph on tourism and its relation to heritage protection and the CCS - your suggestion and explanation was very insightful indeed
- after reading the suggested paper (Gilardi et al. 2023) we have thoroughly revised the section on IP protection
- we have added new references to publications and materials connected with the changes to the paper 
- we have introduced other edits to the paper, following suggestions of Professor Michael Keane who was also kind enough to review the paper and provide his valuable comments.

We are very grateful for this opportunity, hope the paper is a little better now.

# Reviewer report: Reviewer response for version 1

**DOI:** 10.21956/openreseurope.21771.r57868

**Published:** 2025-09-02

**Authors:** Michael Keane

**Affiliations:** 1 Queensland University of Technology, Brisbane, Australia

The paper looks closely at cultural policy in the PRC, namely the outlines of the system, depicted as an organigram. This delegation of policy from the top and its interpretation at local levels will be useful to readers. The paper is well written and covers a lot of issues pertaining to the governance of the traditional cultural policy sphere. The paper largely seeks to map China’s cultural policy initiatives and regulations on to a mostly European policy template. As such, the paper focuses mostly on non-digital production, which is perhaps a legacy of the UNESCO categorisation. In some respects, this doesn’t really capture the state of dynamism across cultural domains in China. See my notes below about the burgeoning digital economy. The data discrepancy on cultural consumption between urban and rural is not surprising although the data classifications are a bit broad. It is hard to calculate per capita online cultural consumption from this.  It is also hard to know how these figures are derived in China.

 The literature review section is okay but probably requires more clarification. The authors have brought together several examples of Chinese and international scholarship in the literature review section. There is in fact a great deal of work on cultural, media and film policy in China, in Chinese, much of which is presented in The Blue Book series published annually by the Chinese Academy of Social Sciences since the late 1990s. However, as the paper notes, there are few if any attempts in existing books or reports to explain the governance system, rather it is about noting policies and speeches, and providing data.  

 It is important to note the Marxist-Leninist relationship pertaining between cultural production and ideology in the PRC from the Yan’an Forum to the 1990s. Culture was a component of the superstructure, which was prioritized in the Cultural Revolution. The early 1990s saw an acceptance of the market mechanism under Deng Xiaoping, the Southern Tour in particular, and this ‘reform of the cultural system’ was extended to public cultural institutions (shiye) which were forced to become industries (chanye/qiye). WTO accession and fear of western culture was thus the driver of marketisation (chanyehua). This theme appears regularly in documents and speeches of the period and is mentioned only in passing once in the paper. As a result of competition, China sought to incorporate cultural models from the west into its system, particularly adding copyright statutes to its system. Scholar-consultants from the west have introduced many ideas over the past two decades.  

 As noted in the paper, the ideological nature of culture is more evident since Xi Jinping came to power i.e. the superstructure/ ideological function has strengthened, and censorship has affected the confidence of many writers and artists, similar to the late Maoist period. As the paper is written from a European perspective it would be useful to note similarities with Soviet cultural policy, notably the emphasis on the Leninist theory of reflection which was subsumed into socialist realism as the template for narrative forms. 

 It is important to mention more about the digital cultural/creative economy and its regulation and governance. This is hinted at on p. 2 with the research question but digital creativity is only really mentioned on p. 12, which is surprising as Chinese people produce and consume culture online, and Chinese culture is now distributed internationally online.  

 The following statement on p. 13 therefore requires more attention “However, it can be assumed that the development of digital technology and the Internet, big data, artificial intelligence and other cutting-edge technologies will have a far-reaching impact on Chinese society and copyright law”. *This is not an assumption*. It is also important to cite Bytedance as a key player in the online video space as well as the predominance of the creator economy, namely the wanghong phenomenon, spurred by Internet + in 2015.  Local governments have sought to monetize the e-commerce/creator economy sector. It is also necessary to note how local governments have incentivized cultural production through clustering of creative workers and the Maker Movement There is a sense that challenges to cultural production are coming thick and fast from AI.

If any, are all the source data and materials underlying the results available?

Yes

Is the work original in terms of material and argument?

Yes

Is the work clearly and cogently presented?

Yes

Is the argument persuasive and supported by evidence?

Yes

Does the research article contribute to the cultural, historical, social understanding of the field?

Yes

Does it sufficiently engage with relevant methodologies and secondary literature on the topic?

Partly

Reviewer Expertise:

Cultural and media policy in China

I confirm that I have read this submission and believe that I have an appropriate level of expertise to confirm that it is of an acceptable scientific standard, however I have significant reservations, as outlined above.

# Response

**Published:** 2025-09-27

**Authors:** Marcin Jacoby

**Affiliations:** Faculty of Humanities, SWPS University of Social Sciences and Humanities, Warsaw, Masovian Voivodeship, Poland

Dear Professor Keane, We are very grateful for your timely and very detalied review of our paper. We have prepared an amended version which, as we hope, includes all the changes and adjustments you proposed:

- we have added a separate paragraph and expanded several other references to the importance and scope of digital cultural content in China
- we have added references to digital culture regulation in China
- we have added a paragraph on creative clusters
- we have mentioned Deng's 1992 Southern Tour and used your interpretation of the additional reasons for market-directed reforms of the cultural sector of the period (WTO, fear of Western culture)
- we have noted parallels between P.R.C.'s understanding of culture and that of the Soviet bloc + added references to Leninism and social realism.

We do hope that the paper looks a little better now!
